# Point-of-care testing diagnosis of African swine fever virus by targeting multiple genes with enzymatic recombinase amplification and CRISPR/Cas12a System

**DOI:** 10.3389/fcimb.2024.1474825

**Published:** 2024-12-04

**Authors:** Shinuo Cao, Dongxue Ma, Jun Xie, Zhi Wu, Haoyu Yan, Shengwei Ji, Mo Zhou, Shanyuan Zhu

**Affiliations:** ^1^ Swine Infectious Diseases Division, Jiangsu Key Laboratory for High-Tech Research and Development of Veterinary Biopharmaceuticals, Engineering Technology Research Center for Modern Animal Science and Novel Veterinary Pharmaceutic Development, Jiangsu Agri-animal Husbandry Vocational College, Taizhou, Jiangsu, China; ^2^ Department of Veterinary Medicine, Agriculture College of Yanbian University, Yanji, Jilin, China; ^3^ MOE Joint International Research Laboratory of Animal Health and Food Safety, MOA Key Laboratory of Animal Bacteriology, College of Veterinary Medicine, Nanjing Agricultural University, Nanjing, Jiangsu, China

**Keywords:** African swine fever virus, point-of-care testing, enzymatic recombinase amplification, CRISPR/Cas12a, diagnosis

## Abstract

African swine fever virus (ASFV) infection is causing devastating outbreaks globally; pig farming has suffered severe economic losses due to the ASFV. Currently, strict biosecurity control measures can mitigate the incidence of ASF. Rapid, cost-effective, and sensitive detection of ASFV can significantly reduce disease transmission and mortality. CRISPR/Cas-associated proteins can detect polymorphisms with high specificity and sensitivity, making them ideal for detecting pathogens. In this study, based on CRISPR/Cas12a integrated with enzymatic recombinase amplification (ERA) technology, a CRISPR/Cas12a detection system capable of identifying ASFV E183L, K205R, and C962R gene sequences has been developed. The ERA-CRISPR/Cas12a detection system detected ASFV precisely without cross-reactivity with other porcine pathogen templates and with a sensitivity detection limit of 10 copies per reaction; it takes 60 minutes to complete the detection process. In combination with this integrated ERA pre-amplification and Cas12a/crRNA cutting assay, it provides a rapid, straightforward, sensitive, and specific method for ASFV detection in the field.

## 1 Introduction

The African swine fever (ASF) virus causes an acute, highly contagious, and systemic febrile disease in pigs (Zurita et al., 2022). The disease is highly infectious and has a high mortality rate, posing a significant threat. As a result, the global pig industry has suffered severe economic losses (Zhao H. et al., 2022; Zhao Y. et al., 2022). Due to the highly complex nature of ASFV’s immune evasion mechanisms and its genetic diversity, there are currently no effective vaccines available for immunoprophylaxis, despite ongoing research efforts. Prevention and control of this disease are challenging due to these factors (Zsak and Neilan, 2002; Zhu et al., 2019; Zhu, 2022; Zhu et al., 2024). Therefore, developing early, precise, and rapid diagnostic techniques for ASFV is particularly important for its biological prevention and control.

In public health and disease prevention, molecular detection technology plays a crucial role (Zhu et al., 2019; Zhao Y. et al., 2022; Zuo et al., 2023). In recent years, outbreaks of ASF have been characterized by increased transmissibility and ability to evade detection, thereby presenting unprecedented challenges to traditional molecular detection methods. Traditional detection techniques, including fluorescence quantitative PCR and isothermal PCR, face inherent limitations (Zsak et al., 2005; Zuo et al., 2021; Zhang et al., 2022; Zhao et al., 2023; Zuo et al., 2023). Fluorescence quantitative and sequencing technologies necessitate sophisticated equipment and skilled personnel, and isothermal PCR, prone to nonspecific amplification, can yield false-positive results. Available nucleic acid detection methods are sensitive and specific, but the detection process is complex and requires expensive instruments, imposing barriers to disease detection. Therefore, researchers hope to develop a set of detection technologies that are straightforward, sensitive, rapid, and inexpensive to address the shortcomings of current technologies.

The integration of enzymatic recombinase amplification with the CRISPR/Cas12a system has shown great promise in the development of point-of-care testing (POCT) methods for various applications. Among them are dengue virus (DENV) (Curti et al., 2020; Mann and Pitts, 2022), avian influenza virus (H7N9) (Zhou et al., 2023), human papillomavirus (HPV) (Zheng et al., 2022; Zhan et al., 2023), Zika virus (ZIKV) (Rahman et al., 2022; Pérez et al., 2024), and SARS-CoV-2 (Liu et al., 2021; Zhang et al., 2021). CRISPR/Cas12a can perform trans cleavage of ssDNA, a feature widely used in nucleic acid detection (Li et al., 2018; Zhang et al., 2023; Zhu et al., 2023). The additional DNA cleavage function of the Cas12a enzyme can cleave specific single-stranded DNA probes (fluorescent and quenched labels) to achieve nucleic acid detection (Chen et al., 2018; Huang et al., 2020; Wang et al., 2020; Liu et al., 2021; Chen et al., 2022). The enzymatic recombinase amplification technique, an isothermal nucleic acid amplification method, can be performed at constant temperatures without the need for thermocycling, and the method is modified from recombinase polymerase amplification (RPA) (Xia and Chen, 2020). In 2018, the Jennifer Doudna’s team combined isothermal amplification technology with CRISPR/Cas12a to develop a detection system called DETECTOR, which can be used for rapid and straightforward real-time detection of trace DNA in samples. In the same year, the HOLMES detection system was published in Cell Discovery, which combines PCR technology with CRISPR/Cas12, requiring no expensive reagents or special instruments, with high efficiency, low cost, and ease of nucleic acid detection (Li et al., 2018).

In this regard, the enzymatic recombinase amplification and CRISPR/Cas12a are integrated in this study to develop a rapid molecular detection system that can efficiently, accurately, and sensitively detect the E183L, K205R, and C962R genes of ASFV. This system provides a convenient and specific detection system for controlling and monitoring ASFV infections in local pig farms.

## 2 Materials and methods

### 2.1 Nucleic acid preparation

The positive standard plasmids pUC57-ASFV-E183L, pUC57-ASFV-K205R, and pUC57-ASFV-C962R were synthesized by GENEWIZ from Azenta Life Sciences (Suzhou, China). The genomic DNA was extracted from pseudorabies live vaccine (Kartha-K61 strain), porcine parvovirus disease live vaccine (WH-1), and inactivated porcine circovirus type 2 vaccine (LG) using a viral DNA extraction kit (Omega, Norcross, GA, USA). The DNA was eluted in an equal volume of elution buffer.

### 2.2 Design and optimization of crRNAs

To target the E183L, K205R, and C962R genes of ASFV genotype II, we designed clustered regularly interspaced short palindromic repeats RNAs (crRNAs) consisting of 20 base pairs positioned immediately downstream of the protospacer adjacent motif (PAM). Each crRNA was tailored to specifically bind to its corresponding gene segment. Following the design phase, the crRNAs were synthesized by GENEWIZ Life Science Company (Suzhou, China). The synthesized crRNAs were then verified for sequence accuracy. The names and core sequences of the different crRNAs are presented in **Table 1**.

**Table 1:** The crRNA, primers, and probes used in this study.

Primers	Sequences (5′-3′)	Size/bp
E183L-forword-1	ATAAATCCTTATCAAGATCAGCAATGGGC	29
E183L-reverse-1	TAGTCTGTCCGTAACTGGGTTGTCCGTGA	29
E183L-crRNA-1	UAAUUUCUACUAAGUGUAGAUUUGCUGGUCUGUUUGUUGCC	41
E183L-forword-2	AAAAGCTGCTGCCGCTATTGAGGAGGAAGATA	32
E183L-reverse-2	TGGTTTGCCTGCACTTGCTGTAGTCGCTCC	30
E183L-crRNA-2	UAAUUUCUACUAAGUGUAGAUGAGGUACCUGGUUGUGGAGU	41
K205R-forword-1	GAGGCTATGCCCCTACCTTCATCAAACACG	30
K205R-reverse-1	GCGTGAAGAACATTGCATTCGTGGGATTTT	30
K205R-crRNA-1	UAAUUUCUACUAAGUGUAGAUACUUGUUUUGCCUGAGGCGG	41
K205R-forword-2	ATCGCCCAACTTGAGATTCTGATGATAAA	29
K205R-reverse-2	GCGTGAAGAACATTGCATTCGTGGGATTT	29
K205R-crRNA-2	UAAUUUCUACUAAGUGUAGAUAUGAAGGUAGGGGCAUAGCC	41
C962R-forword-1	CAATGAATCAGGGAGAAATATGGAAGTGG	29
C962R-reverse-1	ATGGGGCTGACTGAGGTGGTATTTGATGTG	30
C962R-crRNA-1	UAAUUUCUACUAAGUGUAGAUAAGGGUGAUGGACCGAAUCA	41
C962R-forword-2	TAATCATTTTCACGAGCATCCCATTCATC	29
C962R-reverse-2	ATCAAGTTCTGGGATGATGTCTTGGAGTG	29
C962R-crRNA-2	UAAUUUCUACUAAGUGUAGAUAUCCCGAAAACCCCUGGACA	41
ssDNA1	5′-(FAM) TTTTTTTT (BHQ1)-3′	8
ssDNA2	5′-(FAM) TTTATTT (Biotin)-3′	5

### 2.3 Oligonucleotide primers designed for ERA and isothermal amplification

The E183L, K205R, and C962R genes of ASFV were cloned into pUC57 vectors, respectively, and transformed into competent DH5α cells. The ERA primers of ASFV genes (E183L, K205R, and C962R) (**Table 1**) were designed. The isothermal amplification ERA of the E183L, K205R, and C962R gene was performed. Primers for the ERA were synthesized by GENEWIZ Life Science Company (Suzhou, China). The standard ERA reaction was conducted using a basic ERA nucleic acid amplification kit (Suzhou GenDx Biotech Co., Ltd., Suzhou, China). For each sample, a 48 μL premix was prepared, consisting of 20 μL of lysate, 2.5 μL of forward primer (10 μM), 2.5 μL of reverse primer (10 μM), 10 μL of template DNA, and 13 μL of distilled water. This premix was transferred to tubes containing the ERA amplification reagent and mixed thoroughly. Subsequently, 2 μL of ERA activator was added to the lids of the reaction tubes, which were then tightly sealed and briefly centrifuged. The tubes were incubated at 37°C to 42°C for 20 minutes. Finally, the ERA amplification products were analyzed by gel electrophoresis or purified for downstream applications. Following the ERA, the amplified DNA was subjected to a CRISPR/Cas12a cleavage assay to detect the presence of the ASFV genes (E183L, K205R, C962R).

### 2.4 Optimization and evaluation of the CRISPR/Cas12a fluorescence assay

The CRISPR/Cas12a-based detection was conducted in accordance with the experimental protocols outlined in prior research studies (Chen et al., 2018). Briefly, the assay comprised 1 μL of Cas12a enzyme provided by Editgene Co., Ltd. (Guangzhou, China), 3 μL of Cas12a cleavage buffer (10×), 2 μL of crRNA (500 nM), 6 μL of a fluorophore-quencher labeled single-stranded DNA probe, 10 μL of a plasmid containing 10^4^ copies of the pUC57-ASFV gene, and 8 μL ddH2O, totaling a reaction volume of 30 μL. To target specific regions of the ASFV genes E183L, K205R, and C962R, two pairs of specific isothermal amplification primers and two crRNA were designed for each gene. Subsequently, we screened six sets of schemes to identify the most effective combination for the fluorescence assay.

### 2.5 Testing the sensitivity and specificity of the CRISPR/Cas12a fluorescence assay

We assessed the sensitivity of the CRISPR/Cas12a fluorescence assay by performing a 10-fold serial dilution of the standard plasmid, pUC57-ASFV, ranging from 1×10^7^ to1×10^0^ copies per reaction. Double-stranded DNA (dsDNA) plasmid templates were excluded as a negative control. The isothermal amplification was conducted at 37°C for 30 minutes. Subsequent fluorescence assays with these serial dilutions quantified the fluorescent intensity, which was measured under LED blue light illumination. For specificity analysis, the CRISPR/Cas12a fluorescence assay was employed to detect pseudorabies virus (PRV), porcine epidemic diarrhea virus (PEDV), classical swine fever virus (CSFV), porcine circovirus type 2 (PCV2), porcine reproductive and respiratory syndrome virus (PRRSV), *Erysipelothrix rhusiopathiae* (*E. rhusiopathiae*) G4T10 strain, *Mycoplasma hyopneumoniae* (*M. hyopneumonia*e) RM48 Strain and *Pasteurella multocida* (*P. multocida*) 679-230 strain. For ERA amplification aimed at detecting viral genomes, each reaction requires 10 μL of extracted nucleic acid, corresponding to approximately 10 to 100 ng of total nucleic acid.

### 2.6 Rapid immunochromatographic strips preparation

Adapting from an established protocol, we slightly modified the procedure to fabricate a lateral flow detection strip. This strip comprised a sample pad, a conjugate release pad, an absorbent pad, and a nitrocellulose membrane. The conjugate pads, post-blocking buffer application, were dried overnight at 37°C. We employed rabbit anti-mouse IgG for the test line and used a streptavidin conjugate for the control lines, with the nitrocellulose membranes subsequently coated. Gold nanoparticles were utilized for the conjugate pads. The prepared lateral flow strips were stored at 4°C until needed.

### 2.7 CRISPR/Cas12a lateral flow detection

For the lateral flow detection, the 5′ and 3′ ends of the reporter single-stranded DNA (ssDNA) probe were labeled with FAM and biotin, respectively. The CRISPR/Cas12a reaction mixture was diluted in a 1:3 ratio with lateral flow detection buffer. Subsequently, the strips were immersed in this solution and incubated at room temperature for 3 minutes. The results were then documented photographically.

### 2.8 Evaluation of the specificity and sensitivity of lateral flow detection

To assess the sensitivity of the lateral flow detection, standard plasmids pUC57-E183L, pUC57-K205R, and pUC57-C962R were serially diluted in a 10-fold series, respectively, ranging from 1 × 10^7^ to 1 × 10^0^ copies/rxn. A non-template control (lacking dsDNA plasmid) served as the negative control. Isothermal amplification was conducted at 37°C for 30 minutes, followed by dilution of the CRISPR/Cas12a reaction for lateral flow analysis. To evaluate the specificity, we tested the detection system against other swine pathogens, including PRV, PEDV, CSFV, PCV2, and PRRSV.

### 2.9 Integration of triple primer and crRNA sets in a single ERA-CRISPR/Cas12a assay

To enhance the sensitivity of the ERA-CRISPR/Cas12a assay, three primer pairs were concurrently utilized in a single ERA reaction. Subsequently, CRISPR/Cas12a cleavage was executed on the amplification products using three specifically targeted crRNAs.

### 2.10 Statistical analysis

Results are presented as mean values ± standard deviations. Statistical significance was assessed using paired or unpaired t-tests or one-way analysis of variance (ANOVA) with GraphPad Prism 7.0 software. Graphs depict data from a minimum of three independent experiments. The significance levels of P-values were indicated as: ns (not significant); *P ≤ 0.05; **P ≤ 0.01; ***P ≤ 0.001; and ****P ≤ 0.0001.

## 3 Results

### 3.1 CRISPR/Cas12a fluorescence assay validation and optimization

**Figure 1:**
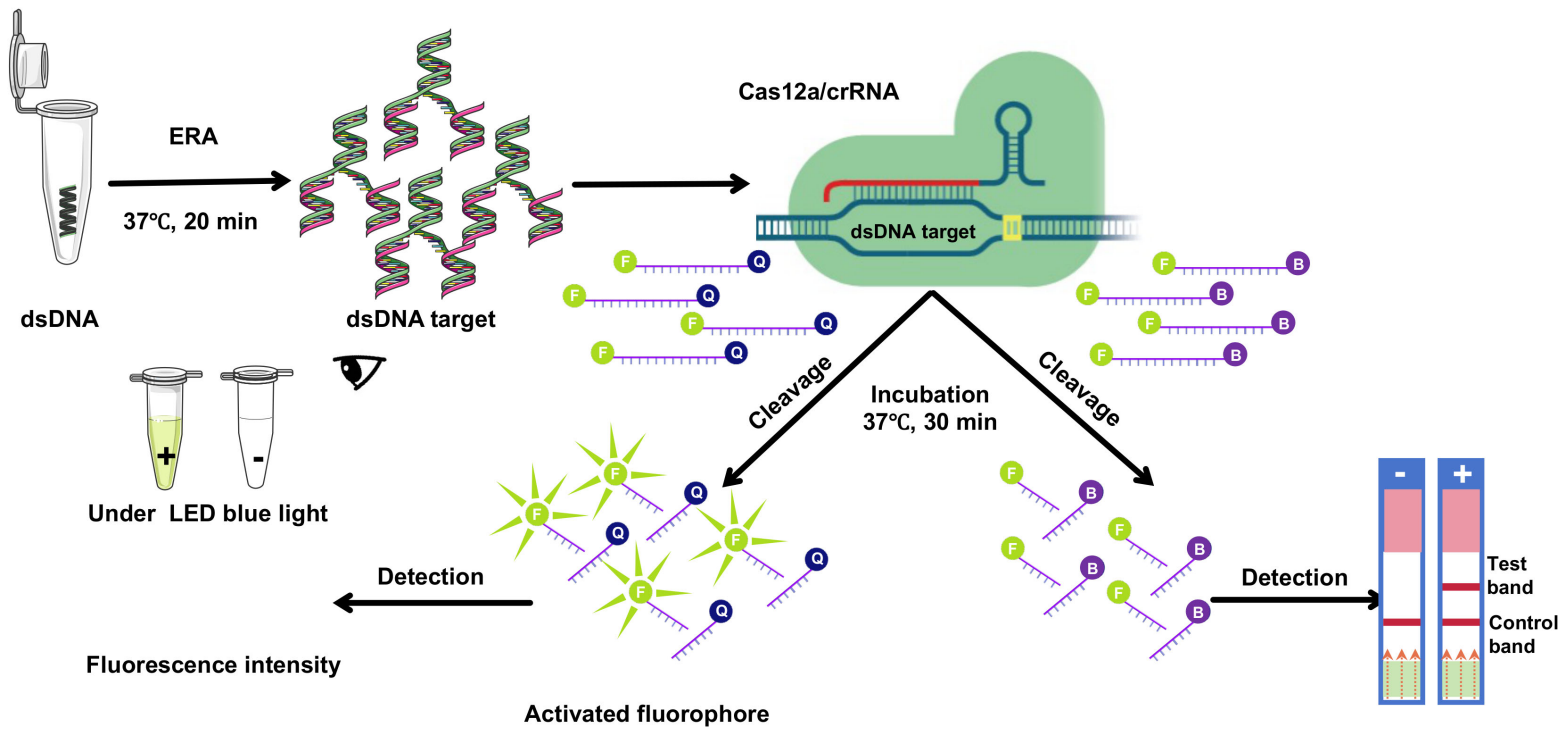
Schematic overview of CRISPR/Cas12a technology integrated with ERA underwent amplification via ERA. Subsequently, the CRISPR/Cas12a complex, guided by crRNA, bound to double-stranded DNA (dsDNA). This interaction facilitated the CRISPR/Cas12a-mediated cleavage of a single-stranded DNA (ssDNA) fluorescent quencher (FQ) probe, where ‘F’ denotes the fluorophore and ‘Q’ the quencher. Fluorescence signals were then detected using suitable equipment and visualized under LED blue light, or through lateral flow immunochromatographic strips.

**Figure 2:**
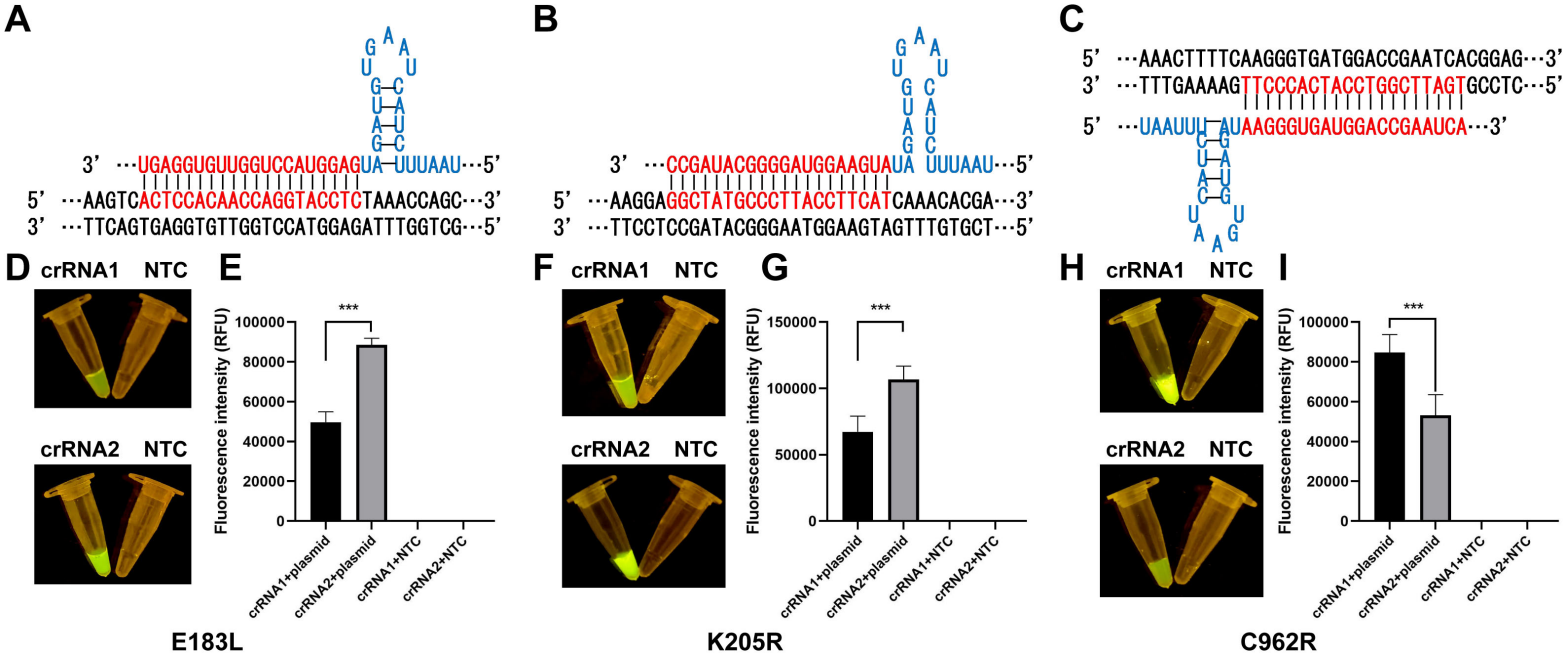
Optimization and validation of crRNAs. **(A)** crRNA-2 targeting of E183L. **(B)** crRNA-2 targeting of K205R. **(C)** crRNA-1 targeting of C962R. **(D, F, H)** Visual inspection under LED blue light. **(E, G, I)** Detection of fluorescence intensity with ABI QuantStudio 5 (n = 3, error bars showed mean ± SEM). Statistical analyses were performed using the student’s t-test in GraphPad Prism. Error bars represent the standard deviation (SD) of the data from three independent experiments. The symbol *** represents that there was a statistical difference (p ≤0.001).

The ERA-CRISPR/Cas12a assay was developed to detect ASFV (**Figure 1**). To assess the efficacy of different crRNAs, we used DNA and negative controls as templates. The ABI QuantStudio 5 system and blue light illumination were employed to visualize the results. We conducted a comparative analysis to determine the most effective crRNA for visual fluorescence detection, using consistent DNA amounts and varying primers. Our findings indicated that the trans-cleavage efficiency of the CRISPR/Cas12a system, induced by E183L-crRNA 2, surpassed that of E183L-crRNA 1; similarly, K205R-crRNA 2 outperformed K205R-crRNA 1, and C962R-crRNA 1 was more effective than C962R-crRNA 2 (**Figure 2**). Consequently, E183L-crRNA 2, K205R-crRNA 2, and C962R-crRNA 1 were identified as well-targeted, exhibiting high fluorescence intensity in detection.

### 3.2 The sensitivity and specificity of CRISPR/Cas12a fluorescence assay

**Figure 3:**
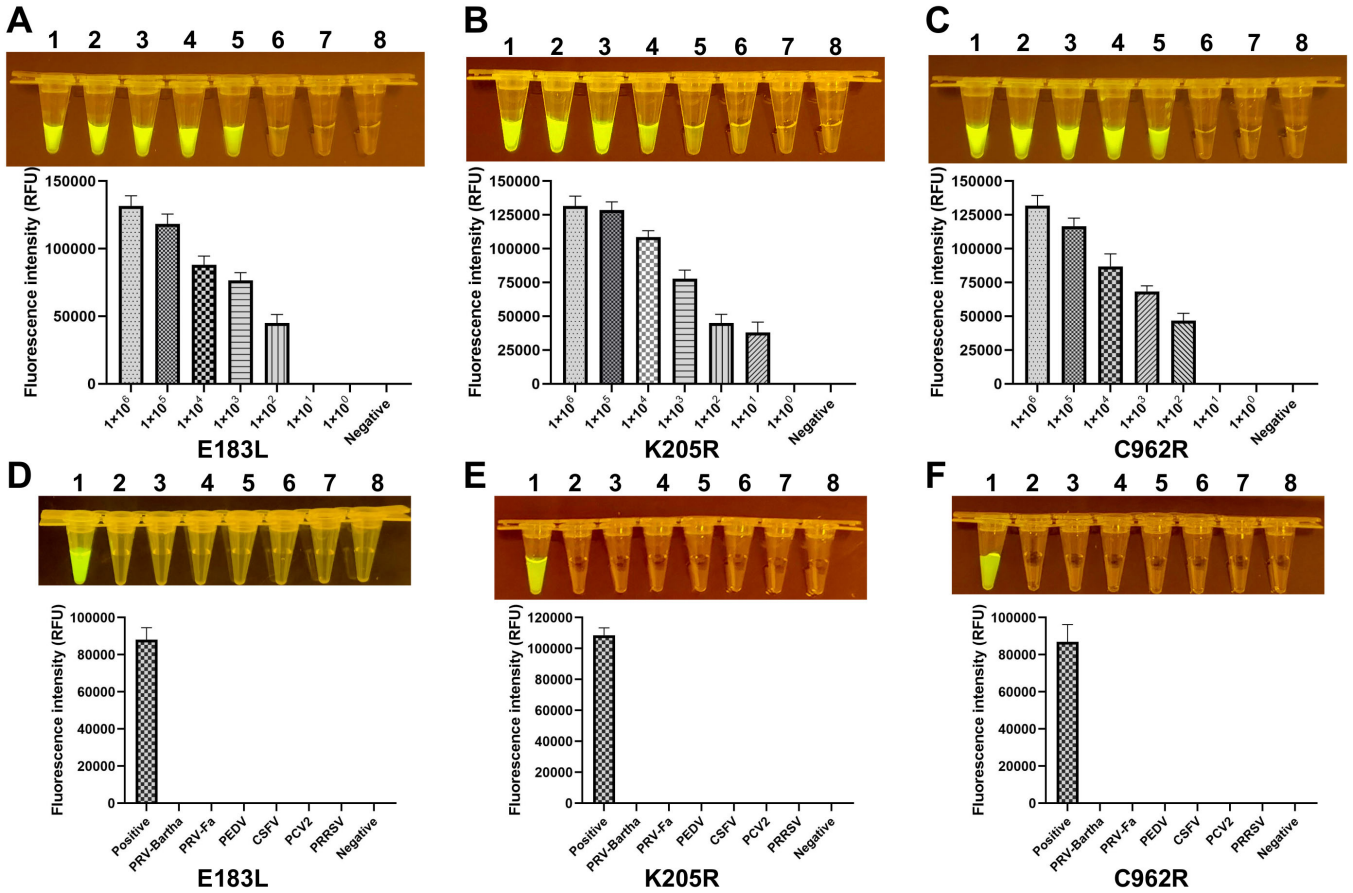
Sensitivity and specificity of the ERA-CRISPR/Cas12a system. **(A-C)** The fluorescence signals obtained from a series of tenfold dilutions of a dsDNA template plasmid, ranging from 1×10^6^ to 1×10^0^ copies/rxn, with a negative control. Each dilution was analyzed using the ERA-CRISPR/Cas12a fluorescence assay, with signal quantification performed by the ABI QuantStudio 5 and visualization by a gel imaging system under UV light. **(D-F)** The ERA-CRISPR/Cas12a specificity for ASFV was measured relative to PRV-Bartha, PRV-Fa, PEDV, CSFV, PCV2, PRRSV, and ddH2O used as the negative control. Error bars represent the standard deviation (SD) of the data from three independent experiments.

To evaluate the sensitivity of the ERA-CRISPR/Cas12a assay, the standard plasmid was serially diluted, and the target fragment was amplified from varying plasmid concentrations. A reciprocal relationship was observed between plasmid concentration and fluorescence intensity, with the minimal detectable limit being 1×10^1^ copies by visual inspection under blue light (**Figure 3**). In the context of clinical samples, which frequently exhibit co-infections with multiple swine viruses, the assay’s specificity was assessed using genomic templates from a range of porcine pathogens, including PRV, PEDV, CSFV, PCV2, PRRSV, *E. rhusiopathia*e, *M. hyopneumoniae* and *P. multocida*. The assay demonstrated exceptional specificity, producing a fluorescent signal exclusively in the presence of ASFV.

### 3.3 Detection of the ASFV gene with CRISPR/Cas12a lateral flow assay

**Figure 4:**
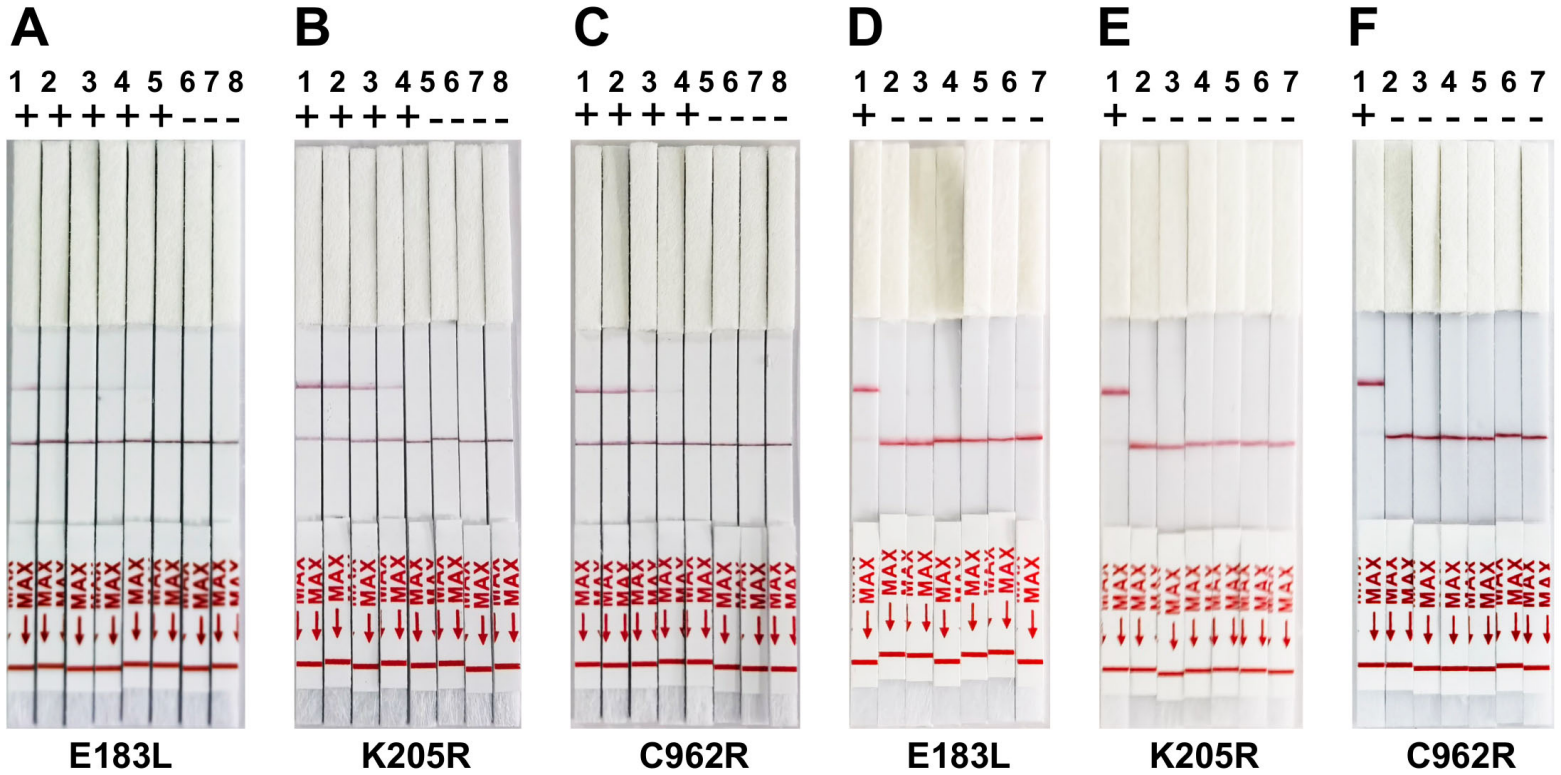
Sensitivity and Specificity of ERA-CRISPR/Cas12a lateral flow detection. **(A-C)** Validation of the sensitivity of CRISPR-Cas12a lateral flow detection. Sensitivity assay using crRNA to detect gradient E183L **(A)**, K205R **(B)**, and C962R **(C)** gene plasmid from 1×10^0^ copies/rxn to 1×10^6^ copies/rxn. 1: 1×10^6^ copies/rxn, 2: 1×10^5^ copies/rxn, 3: 1 ×10^4^ copies/rxn, 4: 1×10^3^ copies/rxn, 5: 1×10^3^ copies/rxn, 6: 1×10^1^ copies/rxn, 7:1×10^0^ copies/rxn, 8: Negative control. **(D-F)** Panels demonstrate the assay’s specificity, showing reactions with ASFV and other swine viruses including PRV-Bartha, PEDV, CSFV, PCV2, and PRRSV. The top band is the test band, and the bottom band is the control band. No color change at the test line was observed for the other swine viruses, indicating specificity of the assay for ASFV. Error bars represent the standard deviation (SD) of the data from three independent experiments.

To assess the sensitivity of the CRISPR/Cas12a lateral flow assay, the pUC57-ASFV plasmid was serially diluted tenfold. Each dilution was tested in triplicate to confirm the assay’s repeatability and stability. The assay successfully detected down to 1 × 10^3^ copies of the ASFV gene. The detection limit for the CRISPR/Cas12a trans-cleavage activity, mediated by E183L-crRNA-B, K205R-crRNA-B, and C962R-crRNA-A, was identified as approximately 1,000 copies of ASFV DNA per reaction (**Figures 4A–C**). Furthermore, the specificity of the CRISPR/Cas12a lateral flow assay for ASFV was evaluated. Through cross-reactivity tests with other swine viruses, it was confirmed that the assay specifically detected ASFV DNA without cross-reactivity, demonstrating its high specificity (**Figures 4D–F**).

### 3.4 Combination of the crRNAs increased the sensitivity of ERA-CRISPR/Cas12a assay

**Figure 5:**
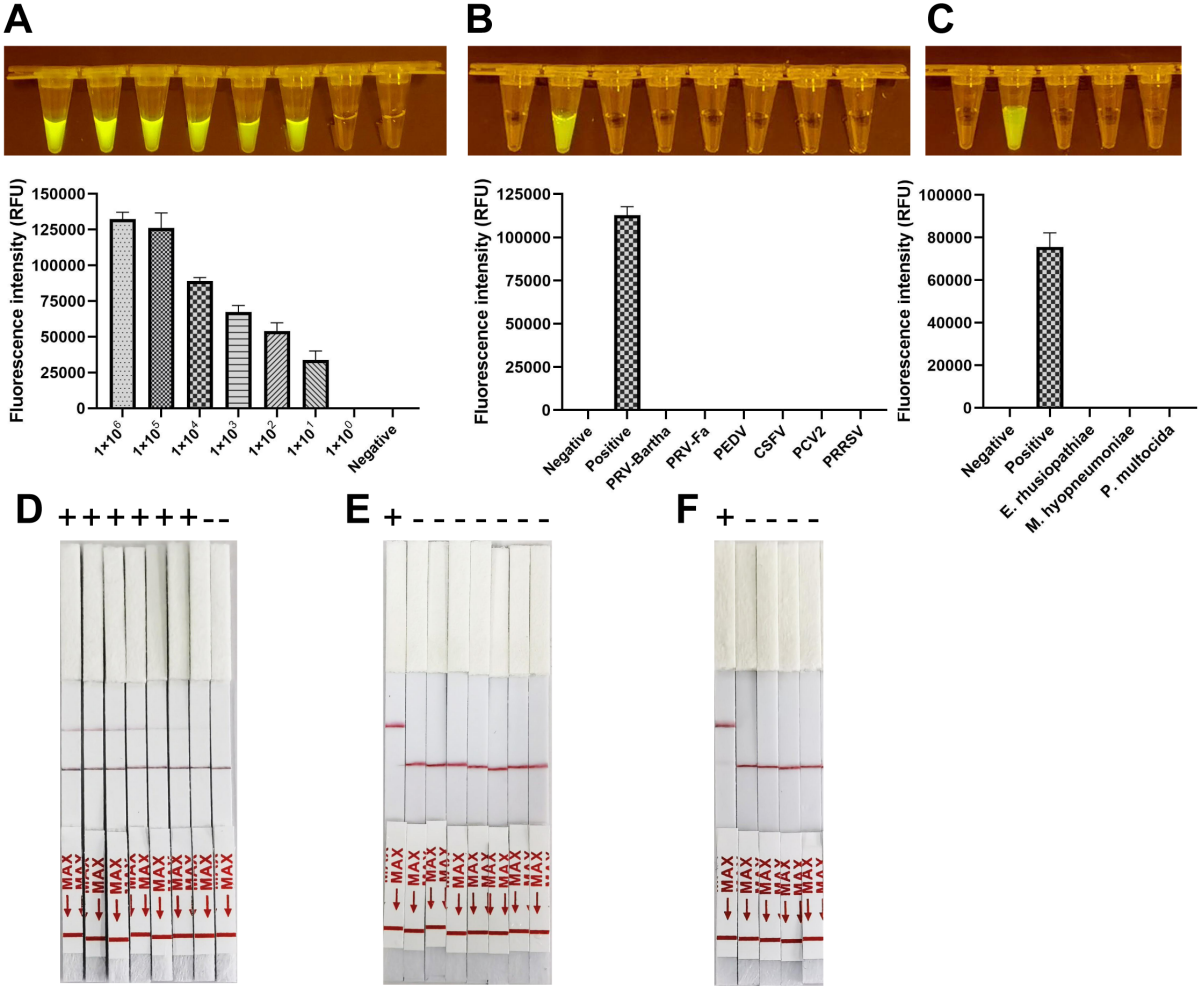
Adapting ERA-CRISPR/Cas12a targeting E183L, K205R, and C962R in the meantime. **(A, D)** The sensitivity of ERA-CRISPR/Cas12a assay targeting multiple genes. The fluorescent signals from a series of 10-fold dilutions of dsDNA template plasmid were detected by the CRISPR-Cas12a fluorescence assay targeting multiple genes and visualized under LED blue light, or through lateral flow immunochromatographic strips. 1: 1×10^6^ copies/rxn, 2: 1 ×10^5^ copies/rxn 3: 1 × 10^4^ copies/rxn, 4: 1 × 10^3^ copies/rxn, 5: 1 × 10^2^ copies/rxn 6: 1 × 10^1^ copies/rxn, 7: 1 × 10^0^ copies/rxn, 8: the negative control. The fluorescent signals produced by different dilutions were calculated by ABI QuantStudio 5. **(B, D)** Determine the specificity of ERA-CRISPR/Cas12a assay targeting multiple by examining ASFV and other porcine pathogens (PRV-Bartha, PRV-Fa, PEDV, CSFV, PCV2, PRRSV, and the negative control). **(C, F)** Determine the specificity of ERA-CRISPR/Cas12a assay targeting multiple by examining ASFV and other porcine pathogens (*E. rhusiopathiae*, *M. hyopneumoniae*, *P. multocida* and the negative control). Error bars represent the standard deviation (SD) of the data from three independent experiments.

To enhance the sensitivity of the ERA-CRISPR/Cas12a assay, three primer pairs were simultaneously utilized in a single ERA reaction. The amplification products, combined with three precisely targeted crRNAs, were subsequently introduced into the CRISPR/Cas12a cleavage assay. This integrative approach achieved a detection limit of approximately 25 copies per reaction, which was further refined to about 20 copies, as demonstrated in **Figure 5**.

## 4 Discussion

African swine fever virus (ASFV) has significantly impacted the swine industry, causing considerable economic losses worldwide due to its rapid transmission. Early diagnosis is crucial to prevent its swift spread (Zuo et al., 2021; Zurita et al., 2022; Zuo et al., 2023). Traditionally, pathogen nucleic acid detection relies on PCR, the gold standard for viral nucleic acid detection (Zuo et al., 2021, 2023). However, PCR requires expensive equipment, skilled personnel, and extended reaction times, rendering it less suitable for rapid, POCT molecular diagnosis of ASFV. For POCT, CRISPR/Cas12a and Cas13a systems are employed, with Cas12a being particularly effective for DNA virus detection. This is because Cas12a can directly interact with isothermal amplification products without requiring transcription (Zhang et al., 2020; Zavvar et al., 2022). Upon recognition of target DNA by the CRISPR-Cas12a-crRNA complex, both cis- and trans-cleavage activities are initiated, leading to the cleavage of the ssDNA reporter in the system (Wang et al., 2022, 2023). The reporter molecule, labeled with a fluorescent group like FAM at the 5’ end and a quenching group like BHQ at the 3’ end, emits fluorescence only upon cleavage. ERA is an isothermal nucleic acid amplification technique that operates at constant temperatures, obviating the need for thermocyclers, thereby facilitating rapid and efficient ASFV detection.

In this research, we developed a Cas12a-based nucleic acid diagnostic assay for detecting ASFV, integrating exponential rolling amplification (ERA) with CRISPR/Cas12a technology. This assay employs crRNA for target specificity and FAM-BHQ as the fluorescent reporter system, with results visualized under blue light. Initially, ERA is conducted, completing the amplification process within 20 minutes. Subsequently, the Cas12a enzyme is introduced to the amplified products, and the mixture undergoes a 30-minute incubation period. The ERA process produces a high quantity of amplified material, enabling the direct visual observation of fluorescence under blue light upon completing the assay.

We developed specific crRNA and primer probes for the ASFV genes E183L, K205R, and C962R to increase amplification efficiency and reduce detection time, thereby enhancing sensitivity. Our optimized system can detect as few as 10 copies of the ASFV K205R gene and 10^0^ copies of the E183L and C962R genes in a 30 µL reaction volume, with the entire process completed within 50 minutes. Cross-reactivity tests with other viruses confirmed the high specificity of our detection system. For practical POCT ASFV diagnosis, we introduced a lateral flow readout mechanism that processes digoxin- and biotin-labeled ssDNA reporters, enabling result visualization on lateral flow strips within 3 minutes. This approach employs nanoparticles for signal amplification, offering benefits such as simplicity, rapidity, low cost, and user-friendliness, obviating the need for specialized training. Consequently, this method is both economical and efficient, requiring no complex instrumentation.

To increase detection sensitivity, we integrated specific crRNA and primer probes targeting the ASFV genes E183L, K205R, and C962R into a single reaction system. This approach markedly enhances the assay’s sensitivity by amplifying three distinct genes simultaneously, thereby augmenting the template availability for the CRISPR/Cas12a detection mechanism. Employing three targeted crRNAs for the ASFV E183L, K205R, and C962R genes intensifies the fluorescence signal, bolstering the detection’s reliability. This multifaceted strategy not only diminishes the likelihood of false positives and false negatives but also facilitates the identification of early-stage infections.

Several studies have employed CRISPR/Cas12a-based systems for point-of-care detection of ASFV. Wang et al. (2020) developed a portable detection method that combined CRISPR/Cas12a with immunochromatographic strips, achieving a detection limit of 1,000 copies per reaction. Zhao H. et al. (2022) utilized a dual CRISPR/Cas12a approach for ASFV p30 antibody detection, demonstrating high specificity. Our ERA-CRISPR/Cas12a system offers substantial advancements, such as enhanced sensitivity (detecting as few as 10 copies per reaction), multiplex gene targeting for improved accuracy, and a rapid, field-deployable format that completes detection in 60 minutes without requiring complex equipment. These characteristics make our assay particularly suitable for early and rapid ASFV detection in resource-limited environments.

## 5 Conclusions

This study developed a highly sensitive and specific method for POCT diagnosis of African swine fever virus (ASFV), targeting multiple genes through enzymatic recombinase amplification (ERA) and the CRISPR/Cas12a system. Our integrated method, combining ERA pre-amplification with Cas12a/crRNA-mediated cleavage, provides a reliable and rapid tool for the diagnosis and differentiation of ASFV, making it well-suited for clinical settings. Early detection afforded by this system facilitates the timely prevention of disease spread, thereby mitigating livestock losses.

## Data Availability

The original contributions presented in the study are included in the article/supplementary material. Further inquiries can be directed to the corresponding author/s.
